# Analysis of the Prognosis and Therapeutic Value of the CXC Chemokine Family in Head and Neck Squamous Cell Carcinoma

**DOI:** 10.3389/fonc.2020.570736

**Published:** 2021-01-08

**Authors:** Yongchao Li, Tinghui Wu, Shujuan Gong, Hangzheng Zhou, Lufei Yu, Meiyan Liang, Ruijun Shi, Zhenhui Wu, Jinpei Zhang, Shuwei Li

**Affiliations:** Key Laboratory of Protection & Utilization of Biological Resources in Tarim Basin, College of Life Sciences, Tarim University, Alar, China

**Keywords:** chemokine, head and neck squamous cell carcinoma, prognosis, bioinformatics analysis, database mining

## Abstract

The CXC chemokines belong to a family which includes 17 different CXC members. Accumulating evidence suggests that CXC chemokines regulate tumor cell proliferation, invasion, and metastasis in various types of cancers by influencing the tumor microenvironment. The different expression profiles and specific function of each CXC chemokine in head and neck squamous cell carcinoma (HNSCC) are not yet clarified. In our work, we analyzed the altered expression, interaction network, and clinical data of CXC chemokines in patients with HNSCC by using the following: the Oncomine dataset, cBioPortal, Metascape, String analysis, GEPIA, and the Kaplan–Meier plotter. The transcriptional level analysis suggested that the mRNA levels of CXCL1, CXCL2, CXCL3, CXCL5, CXCL6, CXCL8, CXCL9, CXCL10, CXCL11, and CXCL13 increased in HNSCC tissue samples when compared to the control tissue samples. The expression levels of CXCL9, CXCL10, CXCL11, CXCL12, and CXCL14 were associated with various tumor stages in HNSCC. Clinical data analysis showed that high transcription levels of CXCL2, CXCL3, and CXCL12, were linked with low relapse-free survival (RFS) in HNSCC patients. On the other hand, high CXCL14 levels predicted high RFS outcomes in HNSCC patients. Meanwhile, increased gene transcription levels of CXCL9, CXCL10, CXCL13, CXCL14, and CXCL17 were associated with a higher overall survival (OS) advantage in HNSCC patients, while high levels of CXCL1, and CXCL8 were associated with poor OS in all HNSCC patients. This study implied that CXCL1, CXCL2, CXCL3, CXCL8, and CXCL12 could be used as prognosis markers to identify low survival rate subgroups of patients with HNSCC as well as be potential suitable therapeutic targets for HNSCC patients. Additionally, CXCL9, CXCL10, CXCL13, CXCL14, and CXCL17 could be used as functional prognosis biomarkers to identify better survival rate subgroups of patients with HNSCC.

## Introduction

Head and neck squamous cell carcinoma (HNSCC) is the sixth most prevalent malignant tumor cancer in the world and has nearly 600,000 new patients each year (1). HNSCC occurs in different locations, including the inner lip, oral cavity, floor of the mouth, larynx, and pharynx. Antibody drugs for the treatment of HNSCC have been developed, such as nivolumab, cetuximab, and pembrolizumab, but the 5-year survival rate is still relatively low for advanced patients (2, 3). Therefore, identification of more accurate prognosis biomarkers and suitable therapy targets are still the primary focus in HNSCC research.

Chemokines are small molecules secreted by cells that play an essential role in chemotaxis and angiogenesis and are moderators of tumor initiation and metastasis (4, 5). There are four families of chemokines, including CXC (17 members), CC (27 members), CX3C (two members), and XC (one member), which are categorized according to the spacing of their first two cysteine residues (6). Chemokines are also classified into two functional groups: homeostatic or inflammatory chemokines. Homeostatic chemokines regulate cell migration and immune surveillance systems and are continuously expressed in normal tissues (7, 8). In contrast, inflammatory chemokines are used to recruit various inflammatory cells to deal with cell damage and infection (9).

CXC cytokines can be divided into ELR+ or ELR- categories according to whether they contain the tripeptide motif Glu-Leu-Arg. Both ELR+ and ELR- chemokines play an essential role in regulating angiogenesis (10, 11). Angiogenesis can promote rapid proliferation and metastasis of tumor cells (12). Therefore, the contributions of distinct CXC chemokines to HNSCC need to be explored. In the present study, we conducted data collection and analyzed the changes in the expression levels of 16 CXC chemokines and their relationship with survival rates based on several large public databases to reveal their potential targets and prognostic values in cancer treatment.

## Materials and Methods

### Oncomine

Oncomine (www.oncomine.org) is a comprehensive online tumor database containing 715 databases and 86,733 samples from 20 types of cancers (13).. We compared the expression differences of each CXC chemokine in tumor and normal samples in 20 types of cancers from the Oncomine database with a threshold limited by P <0.001 and fold >2.

### GEPIA

GEPIA (http://gepia.cancer-pku.cn/) is a powerful online RNA expression analysis tool provides three ways to analyze RNA: single gene analysis, multiple gene analysis, and cancer type analysis. The single-gene analysis module includes gene expression profile analysis, survival analysis, and similar gene detection. Multiple gene comparison, correlation analysis, and principal component analysis are included in the multiple gene analysis module. The cancer type analysis module contains differential expression analysis and the most differential survival gene screening (14).

### cBioPortal Analysis

The cBioPortal (http://www.cbioportal.org/) is a gene annotation and analysis resource for data exploration and analysis of multidimensional cancer genomics and clinic data. The cBioPortal provides more than 60,000 tumor samples. We analyzed the expression and correlation of 16 CXC chemokines in HNSCC by using cBioPortal (TCGA, PanCancer Atlas) (15, 16).

### Functional Enrichment Analysis

Metascape (http://metascape.org) is an online meta-analysis tool that provides expression analysis and custom analysis. Users can use gene annotation, member search, and enrichment analysis in the custom analysis (17). In our study, we performed an enrichment analysis of the CXC chemokine family and 50 neighboring genes. The pathway and process enrichment threshold was limited as follows: min overlap = 3, *p*-value cutoff = 0.01, and min enrichment = 1.5. The protein–protein interaction enrichment threshold was limited as follows: Databases BioGrid + InWeb_IM(human) + OmniPath(human), min network size = 3, and max network size = 500.

### Kaplan–Meier Plotter

The Kaplan–Meier plotter is a line analysis tool that can explore the role of 54,000 genes in the survival of 13,316 cancer samples. These cancer samples include 6,243 breast cancers, 2,190 ovarian cancers, 3,452 lung cancers, and 1,440 gastric cancers (18). We analyzed the correlation between the mRNA expression levels of 16 CXC chemokines and the survival rate of HNSCC by using the Kaplan–Meier plotter.

## Results

### CXC Chemokine Transcriptional Differences in Various Cancers and Normal Tissues

**Figure 1:**
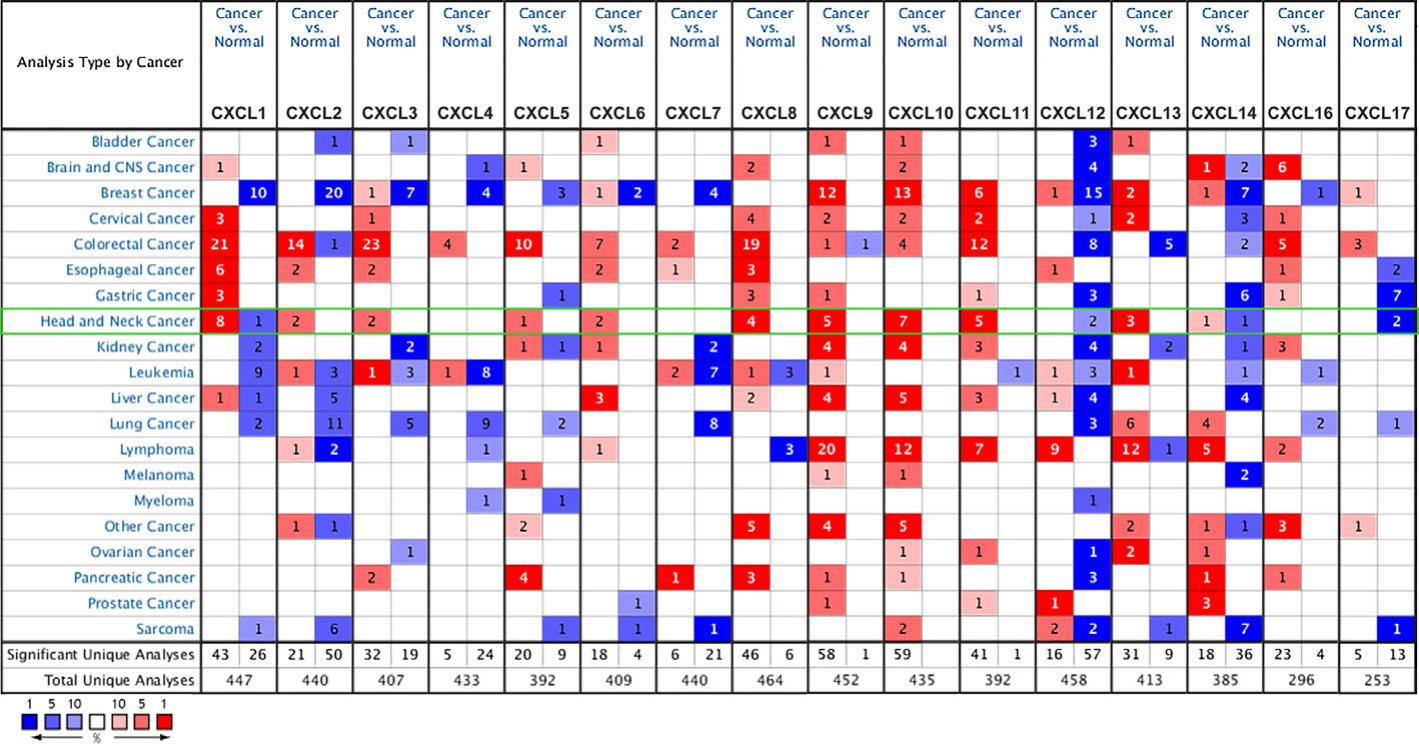
The transcription of CXC chemokines in 20 different types of cancers (ONCOMINE).

We selected 16 chemokines in the CXC chemokine family to measure the differences in their expression in 20 types of cancers and their corresponding normal tissues through the Oncomine database (**Figure 1** and **Table 1**). The results showed that the transcription levels of CXCL1, CXCL2, CXCL3, CXCL5, CXCL6, CXCL8, CXCL9, CXCL10, CXCL11, and CXCL13 in HNSCC samples were significantly higher than those in normal samples. In the Ginos Head-Neck dataset, CXCL1, CXCL2, CXCL3, CXCL5, CXCL6, CXCL8, CXCL9, CXCL10, CXCL11, and CXCL13 were overexpressed in HNSCC samples in contrast with normal tissues, and the fold changes were 17.404, 12.793, 5.374, 16.431, 2.964, 36.405, 6.976, 6.41, 6.635, and 6.901, respectively (19). In the Cromer Head-Neck dataset, CXCL1, CXCL3, CXCL8, and CXCL13 were overexpressed in HNSCC samples in contrast with normal tissues, and the fold changes were 10.047, 2.522, 23.677, and 6.901, respectively (20).

**Table 1:** The significant changes of CXC-chemokine expression in transcription level between different types of HNSCC and normal tissues (oncomine).

	Type	Fold change	T-test	*p*-value	Source and/or Reference	PMID
CXCL1	Head and Neck Squamous Cell Carcinoma vs. Normal	17.404	10.508	4.65E-10	Ginos Head-Neck Statistics	14729608
	Head and Neck Squamous Cell Carcinoma vs. Normal	10.047	4.195	1.00E-02	Cromer Head-Neck Statistic	14676830
CXCL2	Head and Neck Squamous Cell Carcinoma vs. Normal	12.793	8.626	2.53E-08	Ginos Head-Neck Statistics	14729608
CXCL3	Head and Neck Squamous Cell Carcinoma vs. Normal	5.374	7.831	4.25E-10	Ginos Head-Neck Statistics	14729608
	Head and Neck Squamous Cell Carcinoma vs. Normal	2.522	3.656	9.00E-03	Cromer Head-Neck Statistic	14676830
CXCL5	Head and Neck Squamous Cell Carcinoma vs. Normal	16.431	8.134	7.42E-11	Ginos Head-Neck Statistics	14729608
CXCL6	Head and Neck Squamous Cell Carcinoma vs. Normal	2.694	5.557	4.79E-07	Ginos Head-Neck Statistics	14729608
CXCL8	Head and Neck Squamous Cell Carcinoma vs. Normal	36.405	13.407	2.12E-15	Ginos Head-Neck Statistics	14729608
	Head and Neck Squamous Cell Carcinoma vs. Normal	23.677	6.6	1.00E-03	Cromer Head-Neck Statistic	14676830
CXCL9	Head and Neck Squamous Cell Carcinoma vs. Normal	6.976	7.86	1.81E-10	Ginos Head-Neck Statistics	14729608
CXCL10	Head and Neck Squamous Cell Carcinoma vs. Normal	6.41	8.488	1.15E-11	Ginos Head-Neck Statistics	14729608
CXCL11	Head and Neck Squamous Cell Carcinoma vs. Normal	6.635	8.164	3.79E-11	Ginos Head-Neck Statistics	14729608
CXCL13	Head and Neck Squamous Cell Carcinoma vs. Normal	6.901	4.486	3.00E-03	Cromer Head-Neck Statistic	14676830
	Head and Neck Squamous Cell Carcinoma vs. Normal	67.011	14.825	3.47E-16	Ginos Head-Neck Statistics	14729608

### Correlation Between CXC Chemokine Expression Level and Clinicopathological Characteristics of Patients with HNSCC

**Figure 2:**
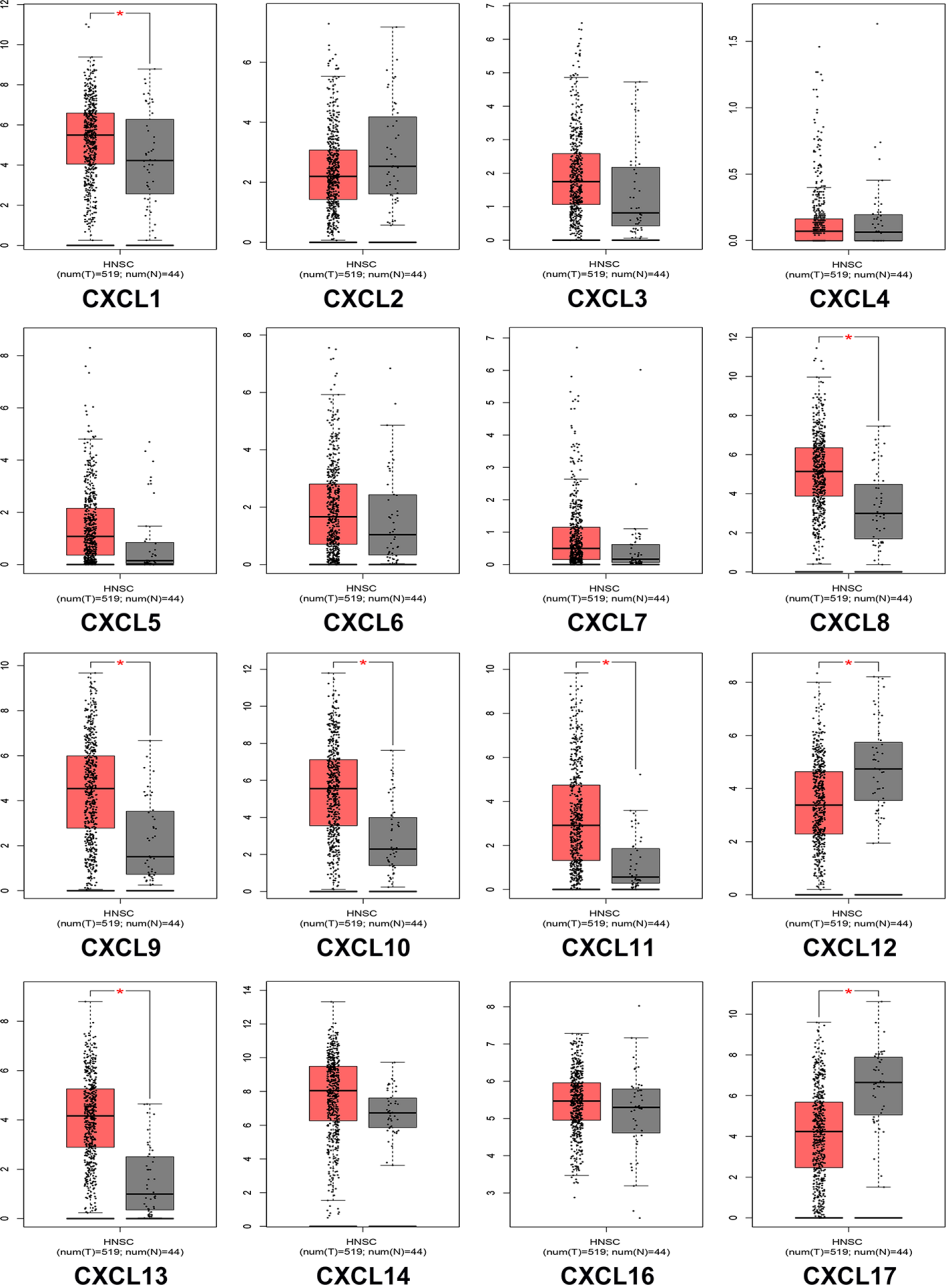
The expression of CXC chemokines in HNSCC patients (GEPIA). The *p*-value = 0.01. * indicated that the results are statically significant.

**Figure 3:**
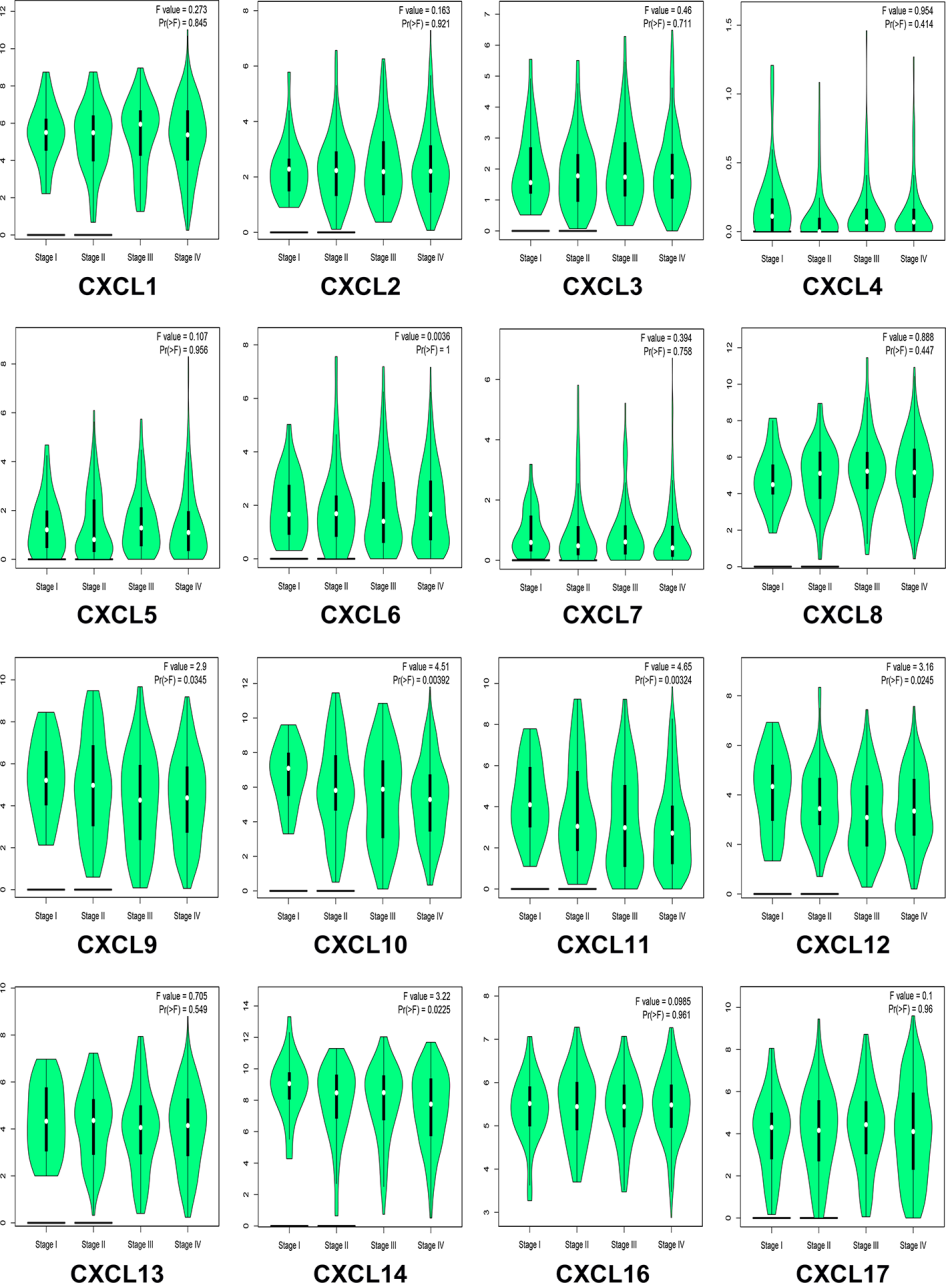
Correlation between CXC chemokines expression and tumor stage in HNSCC patients (GEPIA). the *p*-value was set 0.05.

We analyzed the differences in transcription levels of CXC chemokines in HNSCC and normal samples (**Figure 2**). We found that the transcription levels of CXCL1, CXCL8, CXCL9, CXCL10, CXCL11, and CXCL13 were remarkably upregulated in HNSCC samples when compared to normal samples. However, the transcription levels of CXCL12 and CXCL17 were lower in HNSCC samples than in the normal samples. The relationship among the mRNA levels of distinct CXC chemokines and tumor stages of HNSCC was also explored. The results showed that the CXCL9, CXCL10, CXCL11, CXCL12, and CXCL14 groups varied significantly (**Figure 3**).

### Construction of CXC Chemokine Gene Expression and Protein Interaction Network in Patients With HNSCC

**Figure 4:**
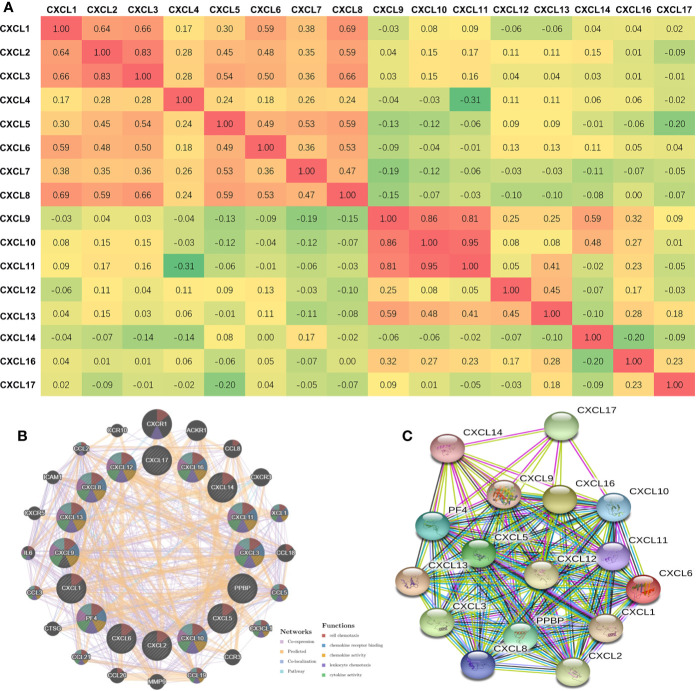
Co-expression and Interaction Analyses of CXCL chemokines at the Gene and Protein Levels in Patients with HNSCC (CBioportal and GeneMANIA and String). **(A)** Pearson correlation of CXC chemokines members. **(B)** Gene-gene interaction network among CXC chemokines members in the CBioportal dataset. **(C)** Protein-protein interaction network among CXC chemokines members in the String dataset.

The Pearson’s correlation of CXC chemokines using expression data (RNA seq V2 RSEM) was calculated by using the cBioPortal online tool for HNSCC samples (TCGA, PanCancer Atlas), and the results showed a clear positive correlation in the following CXCLs: CXCL1 with CXCL2, CXCL3, CXCL6, and CXCL8; CXCL2 with CXCL1, CXCL3, and CXCL8; CXCL3 with CXCL1, CXCL2, CXCL5, CXCL6, and CXCL8; CXCL5 with CXCL3, CXCL7, and CXCL8; CXCL6 with CXCL1, CXCL3, and CXCL8; CXCL7 with CXCL5; CXCL8 with CXCL1, CXCL2, CXCL3, CXCL5, and CXCL6; CXCL9 with CXCL10, CXCL11, and CXCL14; CXCL10 with CXCL9 and CXCL14; CXCL11 with CXCL9 and CXCL10; and CXCL13 with CXCL9 (**Figure 4A**).

We conducted a correlation analysis of 16 CXC chemokines at the gene level through GeneMANIA. Twenty nodes surrounded the 16 central nodes (which represented the 16 CXC chemokines), and showed relationships in pathways, co-expression, predicted, and co-localization. The top five genes that displayed correlations with the CXC chemokine family included CXCR1 (C-X-C motif chemokine receptor 1), ACKR1 (atypical chemokine receptor 1), CCL8 (C-C motif chemokine ligand 8), CXCR3 (C-X-C motif chemokine receptor 3), and XCL1 (X-C motif chemokine ligand 1). CXCR1 was correlated with CXCL12 in terms of co-localization. ACKR1 was connected with CXCL2, CXCL3, CXCL6, CXCL10, CXCL12, CXCL13, and CXCL14 in terms of co-expression and with CXCL12 in terms of co-localization. CCL8 was correlated with CXCL1, CXCL2, CXCL3, CXCL6, CXCL9, CXCL10, CXCL11, and CXCL12 in terms of co-expression. CXCR3 was associated with CXCL3 in terms of co-localization. CXCL1 was correlated with CXCL1, CXCL4, CXCL6, CXCL9, CXCL10, and CXCL13 in terms of co-expression and with CXCL5 and CXCL6 in terms of co-localization. Further functional analysis revealed that these proteins showed the most excellent correlation with cell chemotaxis (FDR = 1.70e^-43^). Additionally, these proteins were correlated with chemokine receptor binding, chemokine activity, leukocyte chemotaxis, cytokine activity, cytokine receptor binding, and G-protein coupled receptor binding (**Figure 4B**). Moreover, through String analysis, we constructed the CXC chemokine protein–protein interaction network to elucidate the interaction between chemokines. The results showed that there are 16 nodes and 111 edges in the protein–protein interaction network (PPI enrichment *p*-value was < 1.0e^-16^), as shown in **Figure 4C**.

### Genetic Alteration of CXC Chemokines and Neighbor Gene Network in Patients With HNSCC

**Figure 5:**
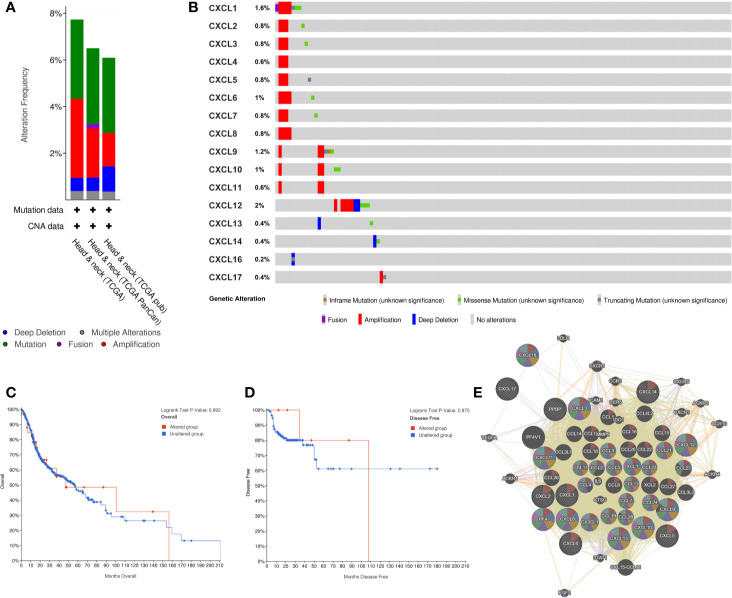
CXC chemokines genetic Alteration and Neighbor Gene Network in patients with HNSCC. **(A)** Summary of alterations in CXC-chemokines members. **(B)** OncoPrint visual summary of alteration on a query CXC-chemokines. **(C, D)** Kaplan-Meier plots comparing OS/DFS in case with/without CXC chemokines member gene alteration. **(E)** Gene-gene interaction network among CXC chemokines members and 50 most frequently altered neighboring genes.

cBioPortal was used to analyze the alteration frequency of CXC chemokine mutations in HNSCC. We examined 1,323 data samples in three HNSCC databases, and the results showed that alterations ranged from 6.09 to 7.74% (**Figure 5A**). The percentages of genetic modification in CXC chemokines for HNSCC ranged from 0.2 to 2% for individual genes, according to the TCGA PanCancer Atlas (**Figure 5B**). Survival results indicated that no noticeable discrepancy in overall survival or disease-free survival exists between the altered group and the unaltered group (**Figures 5C, D**). Then, through GeneMANIA analysis, we constructed the interaction network between CXC chemokines and 50 neighboring genes (**Figure 5E**).

### Gene Ontology and KEGG Kyoto Encyclopedia of Genes and Genomes Pathway Exploration of CXC Chemokines in Head and Neck Squamous Cell Carcinoma Patients

**Figure 6:**
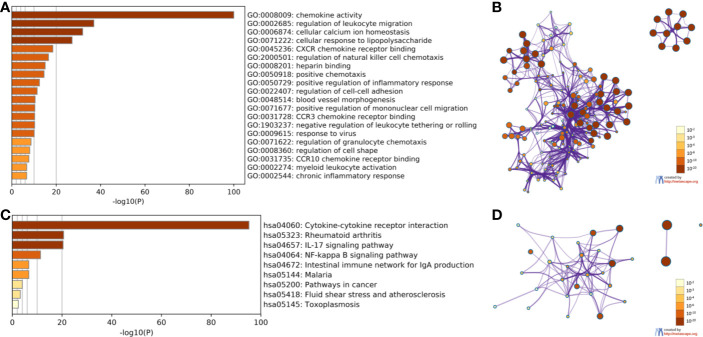
The enrichment analysis of CXC chemokines and neighboring genes in HNSCC (Metascape). **(A)** Heatmap of Gene Ontology (GO) enriched analysis **(B)** Interaction Network of GO enriched analysis. **(C)** Heatmap of Kyoto Encyclopedia of Genes and Genomes (KEGG) enriched analysis. **(D)** Interaction Network of KEGG enriched terms. Note: color by *p*-value.

The Kyoto Encyclopedia of Genes and Genomes (KEGG) and Biological Processes (GO) were analyzed utilizing Metascape to determine the functions of CXC chemokines and their neighboring genes. The enrichment results included 15 biological processes and five molecular function groups (**Figures 6A, B**, and **Table 2**). The biological processes for these genes were mostly leukocyte migration regulation, cellular calcium-ion homeostasis, cellular response to lipopolysaccharides, regulation of natural killer cell chemotaxis, positive chemotaxis, positive regulation of the inflammatory response, regulation of cell-cell adhesion, blood vessel morphogenesis, positive regulation of mononuclear cell migration, negative regulation of leukocyte tethering or rolling, viral response, regulation of granulocyte chemotaxis, regulation of cell shape, myeloid leukocyte activation, and chronic inflammatory response. The molecular functions of these genes were mostly chemokine activity, CXCR chemokine receptor binding, heparin binding, CCR3 chemokine receptor binding, and CCR10 chemokine receptor binding.

**Table 2:** The GO function enrichment analysis of CXC-chemokines and neighbor genes in HNSCC (Metascape).

GO	Category	Description	Count	%	Log10(P)	Log10(q)
GO:0008009	GO Molecular Functions	Chemokine activity	45	68.18	−100	−96.43
GO:0002685	GO Biological Processes	Regulation of leukocyte migration	26	39.39	−36.95	−34.15
GO:0006874	GO Biological Processes	Cellular calcium ion homeostasis	29	43.94	−31.93	−29.21
GO:0071222	GO Biological Processes	Cellular response to lipopolysaccharide	21	31.82	−27.15	−24.55
GO:0045236	GO Molecular Functions	CXCR chemokine receptor binding	8	12.12	−18.48	−16.04
GO:2000501	GO Biological Processes	Regulation of natural killer cell chemotaxis	7	10.61	−16.53	−14.1
GO:0008201	GO Molecular Functions	Heparin binding	13	19.7	−15.05	−12.65
GO:0050918	GO Biological Processes	Positive chemotaxis	10	15.15	−14.53	−12.15
GO:0050729	GO Biological Processes	Positive regulation of inflammatory response	11	16.67	−12.44	−10.11
GO:0022407	GO Biological Processes	Regulation of cell−–cell adhesion	14	21.21	−11.41	−9.12
GO:0048514	GO Biological Processes	Blood vessel morphogenesis	16	24.24	−10.5	−8.22
GO:0071677	GO Biological Processes	Positive regulation of mononuclear cell migration	6	9.09	−10.37	−8.1
GO:1903237	GO Biological Processes	Negative regulation of leukocyte tethering or rolli	4	6.06	−10.29	−8.04
GO:0031728	GO Molecular Functions	CCR3 chemokine receptor binding	4	6.06	−10.29	−8.04
GO:0009615	GO Biological Processes	Response to virus	12	18.18	−10.1	−7.85
GO:0071622	GO Biological Processes	Regulation of granulocyte chemotaxis	6	9.09	−8.67	−6.47
GO:0008360	GO Biological Processes	Regulation of cell shape	8	12.12	−8.11	−5.95
GO:0031735	GO Molecular Functions	CCR10 chemokine receptor binding	3	4.55	−7.71	−5.58
GO:0002274	GO Biological Processes	Myeloid leukocyte activation	12	18.18	−6.75	−4.65
GO:0002544	GO Biological Processes	Chronic inflammatory response	4	6.06	−6.72	−4.62

The KEGG analysis of the CXC chemokines and their neighboring genes revealed nine KEGG pathways (**Figures 6C, D**, and **Table 3**). Among the pathways, those that were involved in HNSCC tumorigenesis and pathogenesis were cytokine-cytokine receptor interaction, the IL-17 signaling pathway, the NF-kappa B signaling pathway, malaria, pathways in cancer, fluid shear stress and atherosclerosis, and toxoplasmosis.

**Table 3:** The KEGG function enrichment analysis of CXC-chemokines and neighbor genes in HNSCC (Metascape).

GO	Category	Description	Count	%	Log10(P)	Log10(q)
hsa04060	KEGG Pathway	Cytokine-cytokine receptor interaction	54	81.82	-95.19	-92.46
hsa05323	KEGG Pathway	Rheumatoid arthritis	14	21.21	-20.64	-18.39
hsa04657	KEGG Pathway	IL-17 signaling pathway	14	21.21	-20.43	-18.3
hsa04064	KEGG Pathway	NF-kappa B signaling pathway	9	13.64	-11.33	-9.5
hsa05144	KEGG Pathway	Malaria	5	7.58	-6.64	-4.99
hsa04672	KEGG Pathway	Intestinal immune network for IgA production	5	7.58	-6.64	-4.99
hsa05200	KEGG Pathway	Pathways in cancer	7	10.61	-4	-2.54
hsa05418	KEGG Pathway	Fluid shear stress and atherosclerosis	4	6.06	-3.21	-1.84
hsa05145	KEGG Pathway	Toxoplasmosis	3	4.55	-2.43	-1.14

### Association of the Transcription Levels of CXC Chemokines With Overall Survival or Relapse-Free Survival of Patients With Head and Neck Squamous Cell Carcinoma

**Figure 7:**
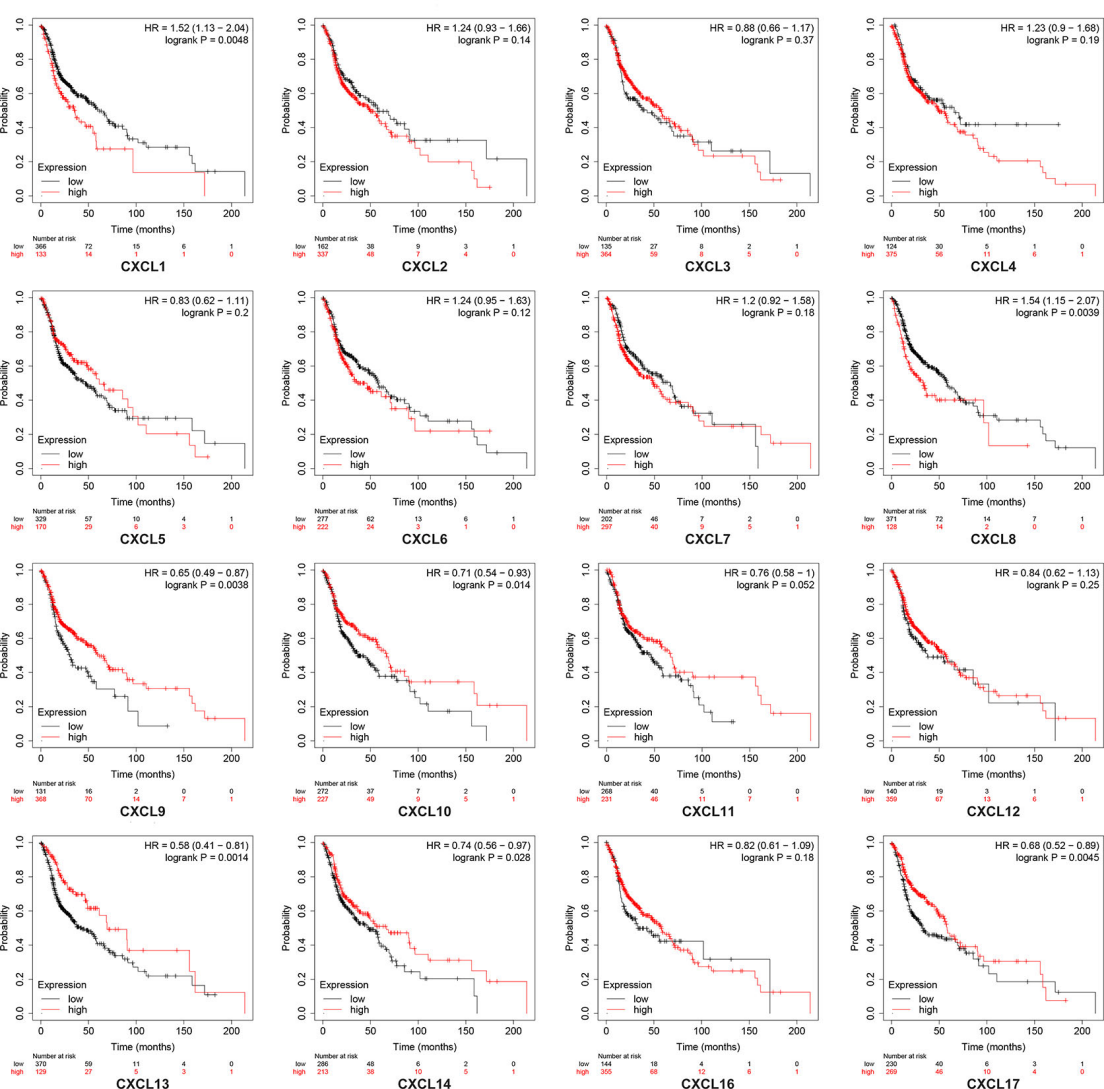
Kaplan-Meier plotter reveals the overall survival differences based on mRNA level of CXC chemokines in HNSCC patients. The threshold of *p*-value of < 0.05.

**Figure 8:**
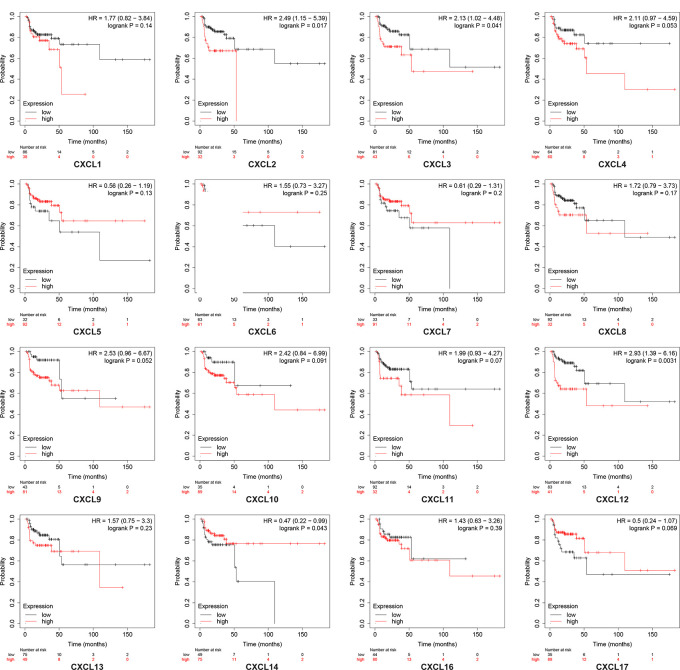
Kaplan-Meier curve reveals the relapse-free survival differences based on mRNA level of CXC chemokines in HNSCC patients.

Using the Kaplan–Meier plotter database, we further explored the relationship among distinct CXC chemokines and the survival rate of HNSCC patients. The results indicated that decreased transcription levels of CXCL1, CXCL8, or the increased transcription levels of CXCL9, CXCL10, CXCL13, CXCL14, and CXCL17 were associated with a long overall survival (OS) outcome (**Figure 7** and **Supplementary Table 1**). Additionally, high mRNA levels of CXCL14 or low mRNA levels of CXCL2, CXCL3, CXCL12 were associated with a better relapse-free survival (RFS) (**Figure 8** and **Supplementary Table 2**).

## Discussion

Recently, more and more studies have pointed out that CXC chemokines are critical regulators of the tumor microenvironment, cancer cell proliferation, and metastasis (12, 21, 22). However, the exact functions of different CXC chemokines in HNSCC is not yet clarified. In this experiment, we identified the expression patterns, prognostic values, and potential functions of different CXC chemokines in HNSCC.

CXCL1 is also named the GRO-1 oncogene (23). Recent studies indicate that CXCL1 is highly expressed in bladder cancer, breast cancer, and prostate cancer (22, 24, 25). In our experiment, GEPIA and Oncomine analysis showed that the expression level of CXCL1 in HNSCC tissues increased significantly when compared to normal tissue. Survival analysis revealed that HNSCC patients with high CXCL1 expression were associated with poor OS outcomes.

CXCL2 is also named Growth-regulated protein beta (Gro-beta), and has 90% amino acid homology with CXCL1 (26, 27). De Filippo et al. (28) confirmed that chemokines CXCL1 and CXCL2 can recruit neutrophils during tissue inflammation. Moreover, Chen et al. (29) found that the CXCL2/CXCR2 axis plays an essential regulatory role in tumorigenesis and cancer stem cell formation. In our study, we identified that the expression levels of CXCL2 in HNSCC tissue increased significantly when compared to normal tissue, and HNSCC patients with high CXCL2 expression were associated with poor RFS outcomes.

CXCL3 is also named the GRO3 oncogene. Earlier studies indicated that the expression of CXCL3 and CXCL8 in colon adenocarcinoma tissue increased significantly when compared with normal tissue (30). Gui et al. (31) also found that CXCL3 is overexpressed in prostate cancer patients. Consistent with previous research, we also found that the transcription levels of CXCL3 in HNSCC samples were remarkably increased when compared with non-tumor tissue. Survival data analysis showed that HNSCC patients with high CXCL3 expression were associated with poor RFS outcomes.

CXCL4, alternatively known as platelet factor 4, was found to be upregulated in various tumoral tissue. Zhang et al. (32) found that the expression of CXCL4 can make tumor cells effectively avoid the monitoring of antitumor immunity, which in turn leads to tumor recurrence and metastasis. Poruk et al. (33) indicated that high serum levels of CXCL4 predicted poor survival in pancreatic cancer patients. In our study, no statistical differences were observed in CXCL4 expression between HNSCC tissues and normal tissues. Interestingly, survival analysis results showed that the transcription levels of CXCL4 are not significantly related to the OS or RFS of HNSCC patients.

CXCL5 is also known as ENA-78 (34), which can bind to CXCR2 to regulate neutrophil homeostasis and promote angiogenesis (35). Benke et al. (36) found that knocking down CXCL5 can reduce the expression of VEGF-C, which can promote the occurrence and metastasis of HNSCC. In this research, we also found that the expression of CXCL5 in HNSCC tissues were significantly increased when compared with non-tumor tissue.

CXCL6 is also named as GCP-2, which can recruit neutrophilic granulocytes (37). Cancer-related research showed that CXCL6 can promote the growth and metastasis of non-small-cell lung cancer (NSCLC) cells *via* down-regulated transcription of MiR-515-5p (38). Ma et al. (39) found that high levels of CXCL6 and CXCL12 synergistically promoted colon cancer metastasis. In our work, we identified that the expression of CXCL6 in HNSCC tissues was significantly upregulated when compared with non-tumor tissue.

CXCL7 is also named pro-platelet basic protein (PPBP), which is a vital regulator in DNA synthesis, glucose metabolism, and the synthesis of hyaluronic acid (40, 41). Tumor-related research revealed that CTAPIII/CXCL7 is an effective diagnostic biomarker for early-stage NSCLC. Desurmont et al. also found the CXCL7/CXCR2 axis as a diagnostic biomarker of poor survival in metastatic colorectal cancer (42, 43). However, in our study, no abnormal expression of CXCL7 was found in HNSCC tissues. Survival analysis results showed that the transcription levels of CXCL17 are not significantly related to the OS or RFS of HNSCC patients.

CXCL8, known as interleukin 8 (IL-8), can combine with the two receptors CXCR1 and CXCR2. Studies indicate that the CXCL8-CXCR1/2 axis can upregulate the expression level of anti-apoptotic proteins in various cancers, thereby promoting the growth, metastasis, and chemoresistance of cancer cells (44). Chan et al. (45) also found that the CXCL8-CXCR1/2 axis promotes HNSCC proliferation by mediating the downstream signaling protein NOD1/RIP. In our study, we observed that the expression of CXCL8 in HNSCC tissues was significantly upregulated when compared with non-tumor tissue. Through survival analysis, we discovered that HNSCC patients with high CXCL8 expression were associated with poor OS.

CXCL9 is also known as MIG, which binds to the CXCR3 receptor. The CXCL9/CXCR3 axis regulates the differentiation and maturation of immune cells (46). Chang et al. (47) research results showed that the serum concentration of CXCL9 in oral squamous cell carcinoma (OSCC) patients can be used as a diagnostic indicator to predict tumor progression and overall survival. In our study, the expression of CXCL9 in HNSCC tissues increased significantly when compared with non-tumor tissue, and the expression was also markedly related to tumor stage in patients with HNSCC. Survival data analysis shows that HNSCC patients with high CXCL9 expression were associated with long OS outcomes.

CXCL10 is also known as small-inducible cytokine B10, which promotes the migration of T cells into inflammatory tissues as well as antitumor activity (48). Chakraborty et al. (49) found that the CXCR3/CXCL10 axis plays an essential function in the regulation of peripheral blood mononuclear cell chemotactic activity with HNSCC patients. In our work, we observed that the transcription of CXCL10 in HNSCC individuals was significantly upregulated when compared with non-tumor tissue, and the mRNA levels of CXCL10 in patients with HNSCC are related to tumor stage. Survival analysis results showed that HNSCC patients with high CXCL10 expression were associated with long OS outcomes.

CXCL11 is also named IP-9. Chemokines CXCL9, CXCL10, and CXCL11 bind to the same ligand CXCR3 (50). However, CXCL11 has a higher affinity with CXCR3 than CXCL9 and CXCL10. Kumaravel et al. (51) found that the CXCR3/CXCL11 axis regulates crosstalk between lymphatic endothelial cells and HNSCC tumors. In this work, we identified that the expression of CXCL11 in HNSCC tissues was remarkably upregulated when compared with non-tumor tissue, and the mRNA levels of CXCL11 in patients with HNSCC were also significantly related to tumor stage.

CXCL12 is also named SDF1. Jin and Yang (52) pointed out the potential value of CXCL12 as a diagnostic marker and a therapy target of HNSCC. Leon et al. (53) also found that low CXCL12 expression levels are significantly related to a high-risk of recurrence of HNSCC. In our study, no abnormal expression of CXCL12 was found in HNSCC tissues. However, CXCL12 expression was found to be markedly related to tumor stage in patients with HNSCC. Survival data analysis showed that HNSCC patients with high levels of CXCL12 were significantly correlated with worse RFS outcomes.

CXCL13 is also named BLC. There are relatively few reports about CXCL13. Sambandam et al. identified that the CXCL13/CXCR5 axis activates the expression of RANKL to prevent OSCC bone metastasis (54, 55). In this study, our analysis results showed that the mRNA levels of CXCL13 in HNSCC tissues were significantly upregulated when compared with non-tumor tissue. Survival analysis results showed that HNSCC patients with high CXCL13 expression were associated with long OS outcomes.

CXCL14 is also known as BRAK. Westrich et al. (56) found that in HPV-positive head and neck cancer cells, CXCL14 can upregulate the expression level of MHC-I to inhibit tumor growth effectively. Kondo et al. (57) also found that the expression level of CXCL14 can be used as a valid diagnostic marker to screen for cetuximab-responsive patients. In this study, no abnormal expression of CXCL14 was found in HNSCC tissues, but CXCL14 expression was found to be markedly related to tumor stage in patients with HNSCC. Survival data analysis showed that HNSCC patients with high CXCL14 levels had a remarkable association with long OS and long RFS.

CXCL16 interacts with the chemokine receptor CXCR6. The mRNA levels of CXCL16 are remarkably increased in breast cancer, prostate cancer, and colon cancer (58). In our study, no abnormal expression of CXCL16 was found in HNSCC tissues. Survival analysis results showed that the transcription levels of CXCL16 are not significantly related to the OS or RFS of HNSCC patients.

CXCL17 is also known as VCC-1 (VEGF co-regulated chemokine 1). Liu et al. (59) found that high expression of CXCL17 in lung adenocarcinoma (LUAD) patients can effectively activate the Src/FAK pathway, thereby promoting the metastasis of LUAD cells. In another study, it was observed that CXCL17 could be used as a new prognosis marker to predict poor OS and RFS in hepatocellular carcinoma patients (60). In our work, the overexpression of CXCL17 was significantly associated with long OS in all patients with HNSCC.

## Conclusions

In summary, our study suggested that CXCL1, CXCL2, CXCL3, CXCL8, and CXCL12 have potential value as new prognosis markers and therapeutic targets for patients with HNSCC, while CXCL9, CXCL10, CXCL13, CXCL14, and CXCL17 could be used as functional prognosis biomarkers to identify better survival rate subgroups of patients with HNSCC. In addition, further experiments are needed to verify the value of CXC family prognosis markers value and their effectiveness as therapeutic targets in patients with HNSCC.

## Data Availability Statement

The datasets presented in this study can be found in online repositories. The names of the repository/repositories and accession number(s) can be found in the article/**Supplementary Material**.

## Author Contributions

SL, YL, and TY conceived the project and wrote the manuscript. SG, HZ, LY, ML, RS, ZW, and JZ participated in the data analysis. All authors contributed to the article and approved the submitted version.

## Funding

This study was supported by the Key Laboratory of Protection and Utilization of Biological Resources in Tarim Basin project (BRZD1906), Tarim University graduate student’s creation project (TDB SCX201905), and National Natural Science Foundation of China (31560685).

## Conflict of Interest

The authors declare that the research was conducted in the absence of any commercial or financial relationships that could be construed as a potential conflict of interest.

## Supplementary Material

Supplementary Table 1 The prognostic value of CXC family members in HNSCC patients is measured by Kaplan-Meier plotter (overall survival).

Supplementary Table 2 The prognostic value of CXC family members in HNSCC patients is measured by Kaplan-Meier plotter (relapse-free survival).

## Abbreviations

HNSCC, head and neck squamous cell carcinoma; OS, overall survival; RFS, relapse-free survival; ENA-78, activating peptide 78; GCP-2, granulocyte chemotactic protein 2; MIG, monokine induced by gamma interferon; IP-9, Interferon-gamma-inducible protein 9; SDF1, stromal cell-derived factor 1; BLC, B lymphocyte chemoattractant; BRAK, breast and kidney-expressed chemokine; VCC-1, VEGF co-regulated chemokine 1.
