# Supplementary material for: Analysis of the Prognosis and Therapeutic Value of the CXC Chemokine Family in Head and Neck Squamous Cell Carcinoma
Source: Front Oncol. 2021 Jan 8;10:570736. doi: 10.3389/fonc.2020.570736 (PMC7820708; doi:10.3389/fonc.2020.570736)
Supplement: Supplementary Table 1 — The prognostic value of CXC family members in HNSCC patients is measured by Kaplan-Meier plotter (overall survival). [file DataSheet_1.docx]

**Supplementary table 1.** The prognostic value of CXC family members in HNSCC patients is measured by Kaplan-Meier plotter (overall survival)

| **CXC family** | **Cutoff value expression** | ***p* -value** | **HR** | **Case** |
| --- | --- | --- | --- | --- |
| CXCL1 | 1545 | 0.14 | 1.77（0.82-3.84） | 124 |
| CXCL2 | 160 | 0.017 | 2.49（1.15-5.39） | 124 |
| CXCL3 | 81 | 0.041 | 2.13（1.02-4.48） | 124 |
| CXCL4 | 1 | 0.053 | 2.11（0.97-4.59） | 124 |
| CXCL5 | 17 | 0.13 | 0.56（0.26-1.19） | 124 |
| CXCL6 | 76 | 0.25 | 1.55（0.73-3.27） | 124 |
| CXCL7 | 2 | 0.2 | 0.61（0.29-1.31） | 124 |
| CXCL8 | 2215 | 0.17 | 1.72（0.79-3.73） | 124 |
| CXCL9 | 756 | 0.052 | 2.53（0.96-6.67） | 124 |
| CXCL10 | 588 | 0.091 | 2.42（0.84-6.99） | 124 |
| CXCL11 | 1767 | 0.07 | 1.99（0.93-4.27） | 124 |
| CXCL12 | 751 | 0.0031 | 2.93（1.39-6.61） | 124 |
| CXCL13 | 520 | 0.23 | 1.57（0.75-3.3） | 124 |
| CXCL14 | 12334 | 0.043 | 0.47（0.22-0.99） | 124 |
| CXCL16 | 1232 | 0.39 | 1.43（0.63-3.26） | 124 |
| CXCL17 | 143 | 0.069 | 0.5（0.24-1.07） | 124 |

Supplementary table 2. The prognostic value of CXC family members in HNSCC patients is measured by Kaplan-Meier plotter (relapse-free survival)

| **CXC family** | **Cutoff value expression** | ***p* -value** | **HR** | **Case** |
| --- | --- | --- | --- | --- |
| CXCL1 | 1826 | 0.0048 | 1.52（1.13-2.04） | 499 |
| CXCL2 | 51 | 0.1413 | 1.24（0.93-1.66） | 499 |
| CXCL3 | 23 | 0.37 | 0.88（0.66-1.17） | 499 |
| CXCL4 | 0 | 0.1912 | 1.23（0.9-1.68） | 499 |
| CXCL5 | 115 | 0.205 | 0.83（0.62-1.11） | 499 |
| CXCL6 | 86 | 0.1166 | 1.24（0.95-1.63） | 499 |
| CXCL7 | 3 | 0.182 | 1.2（0.92-1.58） | 499 |
| CXCL8 | 2441 | 0.0039 | 1.54（1.15-2.07） | 499 |
| CXCL9 | 333 | 0.0038 | 0.65（0.49-0.87） | 499 |
| CXCL10 | 1283 | 0.014 | 0.71（0.54-0.93） | 499 |
| CXCL11 | 246 | 0.052 | 0.76（0.58-1） | 499 |
| CXCL12 | 204 | 0.25 | 0.84（0.62-1.13） | 499 |
| CXCL13 | 781 | 0.0014 | 0.58（0.41-0.81） | 499 |
| CXCL14 | 12875 | 0.028 | 0.74（0.56-0.97） | 499 |
| CXCL16 | 1146 | 0.18 | 0.82（0.61-1.09） | 499 |
| CXCL17 | 0.0045 | 0.0045 | 0.68（0.52-0.89） | 499 |
